# Modifiable risk factor profiles moderate the effect of β-amyloid pathology on cognition in aging

**DOI:** 10.1016/j.neurobiolaging.2025.05.001

**Published:** 2025-05-15

**Authors:** Mohini Bhade, Stefania Pezzoli, Joseph Giorgio, Tyler J. Ward, Joseph R. Winer, Theresa M. Harrison, Susan M. Landau, William J. Jagust

**Affiliations:** a Department of Neuroscience, University of California, Berkeley, Berkeley, California, USA; b Molecular Biophysics and Integrated Bioimaging, Lawrence Berkeley National Laboratory, Berkeley, California, USA; c School of Psychological Sciences, College of Engineering, Science and the Environment, University of Newcastle, Newcastle, New South Wales, Australia; d Stanford University School of Medicine, Stanford, California, USA

**Keywords:** Resilience, Normal aging, Cognition, Lifestyle, Risk factor, B-amyloid, Tau

## Abstract

Although modifiable risk factors may account for around 40 % of population variability in dementia risk, the effect of risk factor interrelationships on pathology-cognition relationships is poorly understood. Using risk factor data from a cohort of 203 cognitively normal older adults (73 ± 6.4 years, 56 % female), we used k-means clustering to assign participants to one of three risk-related profiles; namely, positive-active (physical/cognitive activity, education), positive-affective (sleep, depression, personality), and negative multi-domain clusters. Linear mixed-effects models showed an attenuated effect of β-amyloid on non-memory cognition decline in positive profiles (positive-active: β=3.7, p = 0.008, positive-affective: β=3.7, p = 0.007) compared to the negative profile. While a significant entorhinal tau x time effect (p <0.001) was observed in a model predicting episodic memory decline, cluster membership did not modify this relationship. These findings suggest that different risk profiles moderate pathology-cognition relationships, and highlight the role of groups of modifiable resilience factors in mitigating the effects of β-amyloid deposition.

## 1. Introduction

Around 40 % of the population variability in risk of developing dementia, including Alzheimer’s disease (AD), has been epidemiologically attributed to modifiable risk factors (Livingston et al., 2020; Ourry et al., 2023). A life-course understanding of how risk factors interact with AD pathophysiology will therefore be crucial in developing new therapeutics and interventions. There are two main pathways through which modifiable risk factors have been shown to influence AD risk (Ourry et al., 2023). In the resistance pathway, modifiable risk factors delay the appearance and accumulation of AD pathology (i.e. β-amyloid (Aβ) or tau accumulation) before the onset of cognitive impairment (Ourry et al., 2023). In the resilience pathway, often operationalized as cognitive reserve, modifiable risk factors may function as effect modifiers that lessen the effects of AD pathology on cognition (Ourry et al., 2023; Stern et al., 2023).

Factors such as cardiovascular disease risk (Rabin et al., 2018), physical activity (Rabin et al., 2019), education (Bennett et al., 2003; Ko et al., 2022; Litke et al., 2021), cognitive activity (Ko et al., 2022), sleep (Wilckens et al., 2018; Winer et al., 2019; Zavecz et al., 2023), personality (Strikwerda-Brown et al., 2022; Yoon et al., 2020), and psycho-affective disorders (Moon et al., 2017) all play a role in both pathways; however, conflicting evidence complicates definitive conclusions. For instance, Framingham Risk Score (FRS), physical activity levels, education, and lifetime cognitive activity have all been linked to cognitive decline in relationships that are both independent and synergistic with AD pathology (Bennett et al., 2003; Ko et al., 2022; Litke et al., 2021; Rabin et al., 2019, 2018). On the other hand, personality traits like conscientiousness as well as changes in sleep quantity and electrophysiological quality have been shown to directly protect against accumulations of AD pathology (Wilckens et al., 2018; Winer et al., 2019; Yoon et al., 2020). Other authors, meanwhile, have noted the likely bidirectionality of the relationships between sleep and AD pathology, and the limitations of discussing sleep as a risk factor (Huang et al., 2020; Lucey, 2020; Minakawa et al., 2019; Wang and Holtzman, 2020). Neuropsychiatric symptoms and disorders, including depression, have similarly been implicated in primarily bidirectional associations with AD pathology and cognition (Canevelli et al., 2013; Huang et al., 2020; Kuo et al., 2020; Liew, 2020; Moon et al., 2017; Royall and Palmer, 2013). Ultimately, while these factors interplay in complex ways, a multifaceted approach is crucial in understanding how modifiable risk interacts with cognitive decline along AD pathways.

The bulk of these previous studies have investigated single AD risk factors while statistically controlling for other lifestyle modifiers (i.e. education). However, even with increasing evidence that multiple modifiable risk factors likely cluster together (Conry et al., 2011; LaPlume et al., 2022), there is still limited data regarding the contribution of multi-domain risk factor occurrence on AD-related cognitive decline and AD pathology-cognition relationships. Emerging work has shown that adults with high risk in multiple modifiable risk factor domains show worse Preclinical Alzheimer Cognitive Composite (PACC) scores, worse executive function composite scores, increased subjective cognitive complaints, and increased levels of AD pathology compared to individuals displaying no risk or risk in a single domain (Bransby et al., 2024, 2023). The Lifestyle for Brain Health (LIBRA) score, a multidimensional lifestyle-based dementia risk score, has also been associated with greater cognitive impairment and decline across different tests (Cody et al., 2022; Heger et al., 2021). Considering these reports, we posit that modifiable AD risk factors are highly intercorrelated and risk factor interrelationships convey important information regarding total lifetime advantage and broader socio-environmental effects that should themselves be studied as drivers along the AD pathway.

Therefore, in this study we aim to investigate the combined effect of lifestyle and personality risk factors as a whole on the AD pathway. From these analyses, we hope to not only develop novel insights regarding possible lifestyle interventions, but also focus attention on the upstream determinants of AD resistance and resilience mechanisms.

## 2. Methods and materials

### 2.1. Participants

Participant data was obtained from the longitudinal, observational Berkeley Aging Cohort Study (BACS). All BACS participants are cognitively normal older adult volunteers who were recruited into the cohort via advertisements and word of mouth. Inclusion criteria for BACS include a Mini-Mental State Examination (MMSE) score ≥ 25, normal daily function, and scores on the California Verbal Learning Test (CVLT) and Visual Reproduction tests within 1.5 standard deviations of age, sex, and education-adjusted norms. Exclusion criteria include history of neurological disease or substance abuse, cognition-affecting mental illness, or neuroimaging contraindications. Individuals with cognitive decline suggesting clinically relevant impairment were excluded so that none had mild cognitive impairment. In this study, we examined 203 participants who were aged 60 and older who had completed lifestyle, sleep, personality, and neuropsychological tests. Additional requirements for the pathology cohort were completed [11 C]Pittsburgh Compound B (PiB) positron emission tomography (PET) scans to measure Aβ pathology (n = 149), and [18 F] flortaucipir (FTP) PET scans to measure tau pathology (n = 126). Composite scores for episodic memory and non-memory cognition were generated using confirmatory factor analyses of neuropsychological data (Dobyns et al., 2023).

The University of California, Berkeley and Lawrence Berkeley National Laboratory (LBNL) institutional review boards have approved all study protocols. All participants provided written, informed consent for their participation in the study.

### 2.2. Neuropsychological assessment and composites

The BACS protocol encompasses a comprehensive neuropsychological assessment battery that evaluates various cognitive domains, including verbal and visual memory, working memory, processing speed, executive functioning, and attention. This study utilized the following tests: CVLT, Logical Memory, Visual Reproduction, Trail Making Test (TMT) A and B, Stroop test, digit symbol task, phonemic verbal fluency F-A-S test, Animal Naming, Vegetable Naming, Digit Span Forward and Digit Span Backward, and Boston Naming Test. Only participants who underwent a full neuropsychological battery assessment were included in the study. Additionally, 86 % of participants in our cohort had repeated neuropsychological assessments available, with 175 out of 203 participants having completed two to fourteen sessions.

Dobyns et al. extensively detailed the methodology for calculating episodic memory (EM) and non-memory cognition (NM) composite scores using Confirmatory Factor Analyses (CFAs). The EM composite score was derived from tests including CVLT Short Delay Free Recall (SDFR), CVLT Long Delay Free Recall (LDFR), Visual Reproduction I, Visual Reproduction II, Logical Memory Total Score, and Verbal Paired Associates (Dobyns et al., 2023). Meanwhile, the NM composite, largely referring to executive function, encompassed tests such as Stroop in 60 s, Digit Symbol, TMT-A, TMT-A subtracted from TMTB (Trails B–A), Digit Span Backward, Animal Naming, and Vegetable Naming (Dobyns et al., 2023).

### 2.3. K-means clustering

We conducted k-means clustering in R version 4.2.3 using the *factoextra* (Kassambara, Mundt, 2020) and *cluster* (Maechler et al., 2022) packages to segment all participants into different groups based on lifestyle and personality risk factor data. All longitudinal sessions where lifestyle, personality, cognitive, and sleep testing dates were matched within 1 year of each other were used as data points (i.e. 487 data points from 203 participants), and principal component analysis (PCA) was used to visualize cluster boundaries. The variables used in the clustering were:

- Years of education: obtained from participant demographic data.
- Physical activity: assessed using a formula (Siscovick et al., 1997) that converted lifestyle questionnaire responses into total kCal/week.
- Framingham Risk Score (FRS): generated by a validated formula (D’Agostino et al., 2008) that utilized the following cardiovascular risk factors as input variables: body mass index (BMI), systolic blood pressure, blood pressure lowering medications (yes/no), age, sex, diabetes type II status (yes/no), and ever smoking status (yes/no). Cardiovascular risk factor data were obtained from participant medical histories collected during lifestyle testing.
- Late life cognitive activity: assessed using the Lifetime of Experiences Questionnaire (LEQ) Late Life score (Valenzuela and Sachdev, 2007) collected during neuropsychological testing.
- Depression: as indicated by the total Geriatric Depression Scale (GDS) score (Yesavage et al., 1982) collected during neuropsychological testing.
- Big 5 Personality Traits: as indicated by the Big Five Inventory (Agreeableness, Openness, Conscientiousness, Extraversion, and Neuroticism) scores (John et al., 1991) collected during neuropsychological testing.
- Sleep quality: as indicated by the global Pittsburgh Sleep Quality Index (PSQI) score (Buysse et al., 1989) collected during neuropsychological testing.

Following assignment of each lifestyle session to a cluster, participants were assigned to a “most common cluster” by determining which cluster they were assigned to in the majority of sessions. Ties in cluster assignment (19/203 participants) were broken using a random sampler function in R. A large majority of participants (77 %, 156/203) remained in the same cluster through all longitudinal sessions. Thus, lifestyle risk was operationalized as a trait value, which in turn allowed us to study its effect on overall cognitive trajectory.

### 2.4. PET acquisition and processing

149/203 participants underwent Aβ-PET imaging with PiB, and 126/203 participants underwent tau-PET imaging with FTP using a BIOGRAPH PET/CT scanner following established protocols outlined in prior publications (Ossenkoppele et al., 2016; Schöll et al., 2016; Villeneuve et al., 2015). PiB and FTP tracers were synthesized at the LBNL Biomedical Isotope Facility. PiB-PET images were acquired with 90 min of dynamic emission data frames post-injection of 15 mCi of PiB tracer, with attenuation correction performed using a pre-injection computerized tomography (CT) scan. Reconstruction utilized an ordered subset expectation maximization algorithm with weighted attenuation and a 4-mm Gaussian kernel smoothing with scatter correction. The distribution volume ratio (DVR) was calculated using Logan graphical analysis on PiB frames acquired between 35 and 90 min post-injection and then standardized with reference to the cerebellar gray matter (Logan et al., 1996; Price et al., 2005). Global cortical PiB DVR was calculated using FreeSurfer-derived cortical regions of interest (ROIs) (Desikan et al., 2006; Mormino et al., 2011), with Aβ positivity determined using a global PiB DVR threshold of 1.065 (Villeneuve et al., 2015).

For FTP-PET images, participants received 10 mCi of tracer and were scanned 80–100 min post-injection, usually on the same day as the PiB-PET scan. Reconstruction utilized an ordered subset expectation maximization algorithm with scatter correction and 4-mm Gaussian kernel smoothing. Standardized uptake value ratio (SUVR) images for FTP were created by normalizing mean tracer uptake to the inferior cerebellar gray matter reference region (Baker et al., 2017a). Geometric transfer matrix partial volume correction (PVC) on Desikan-Killiany FreeSurfer-derived ROIs was applied for FTP data processing to address partial volume effects (Baker et al., 2017b; Rousset et al., 1998). The entorhinal cortex (EC) ROIs defined by the Desikan-Killiany atlas were specifically utilized to explore differences in FTP uptake between clusters because this region encompasses the transentorhinal cortex, the region of earliest cortical tau deposition (Braak and Braak, 1995, 1992; Chen et al., 2021). Similarly, the metatemporal ROI (MetaROI), encompassing the entorhinal cortex, the parahippocampal, inferior temporal, the middle temporal and fusiform gyri and the amygdalae, was utilized to explore between-cluster differences in FTP uptake because this is one of the earliest regions of tau spread in aging and early AD (Braak and Braak, 1991).

### 2.5. Statistical analyses

All statistical analyses were carried out in R version 4.2.3.

Demographic variables were compared between cluster cohorts using one-way analysis of variance (ANOVA) tests for continuous variables and Fisher’s exact tests for categorical variables. Post-hoc Tukey’s honest significant difference (HSD) tests were used to assess between-cluster differences for continuous variables. Cross-sectional analyses of age- and sex-controlled differences in cognition and pathology between clusters were conducted using type III ANOVA tests with the *multcomp* (Hothorn et al., 2008) and *car* (Fox, Weisberg, 2019) packages.

Linear mixed-effects models (LMEMs) with random intercept and slope were generated using the *lmerTest* (Kuznetsova et al., 2017) and *sjPlot* (Lüdecke, 2022) packages and were used to assess associations between cluster assignment and decline in EM and NM. All models included age at baseline and sex as control covariates, and random effects of subject intercept and time slope. Time was operationalized as years from baseline cognitive session for each participant. To operationalize pathology measures in a manner most likely to capture salient aspects of participants on the AD pathway, we defined the variables “PiB-status”, “EC tau”, and “MetaROI tau” and added them to linear mixed effects models to determine if there were modulatory effects of cluster on the relationship between Aβ or tau pathology, respectively, and cognitive decline. For the 149/203 participants with one or more longitudinal amyloid scans, positive PiB-status was defined as the presence of ≥ 1 positive amyloid scans (model 2). For the 126/203 participants with available FTP scans, EC and MetaROI tau were defined as the maximum entorhinal or MetaROI FTP uptake, respectively, recorded in longitudinal scans (model 3). The models are outlined below.

- *Model 1*: Cognition (EM or NM) ~ time + cluster*time + baseline age + sex + (1 + time | participant)
- *Model 2*: Cognition (EM or NM) ~ time + cluster*time + PiB-status*time + cluster*PiB-status*time + baseline age + sex +(1 + time | participant)
- *Model 3*: Cognition (EM or NM) ~ time + cluster*time + EC or MetaROI tau*time + cluster*EC or MetaROI tau*time + baseline age + sex + (1 + time | participant)

#### 2.5.1. Sensitivity analyses

We conducted sensitivity analyses to test the fidelity of the observed moderating effect of cluster on the relationship between Aβ pathology and NM decline. First, we added either EC tau or APOE ε4 carrier status as an additional covariate in model 2 to evaluate if the observed moderating effect was dependent on genetic AD risk or tau pathology. As well, we replicated our model 2 analyses substituting maximum PiB-DVR, a measure reflecting the highest global PiB-DVR from a participant’s longitudinal scans, for PiB-status to assess if there were differences when employing a continuous versus categorical measure of Aβ pathology.

We additionally used the following LMEM to investigate whether cluster assignment may moderate the relationship between PiB-status and longitudinal EC tau accumulation: EC tau ~ time + cluster*time + PiB-status*time + cluster*PiB-status*time + baseline age + sex + (1 + time | participant). Note here that time refers to years from the FTP-PET scan baseline for each participant. This was done in order to determine whether the observed between-cluster differences in non-memory cognition decline amongst Aβ-positive individuals may be related to differential Aβ-EC tau relationships.

## 3. Results

### 3.1. K-means clustering reveals patterns of risk factor interrelationships

**Fig. 1. |:**
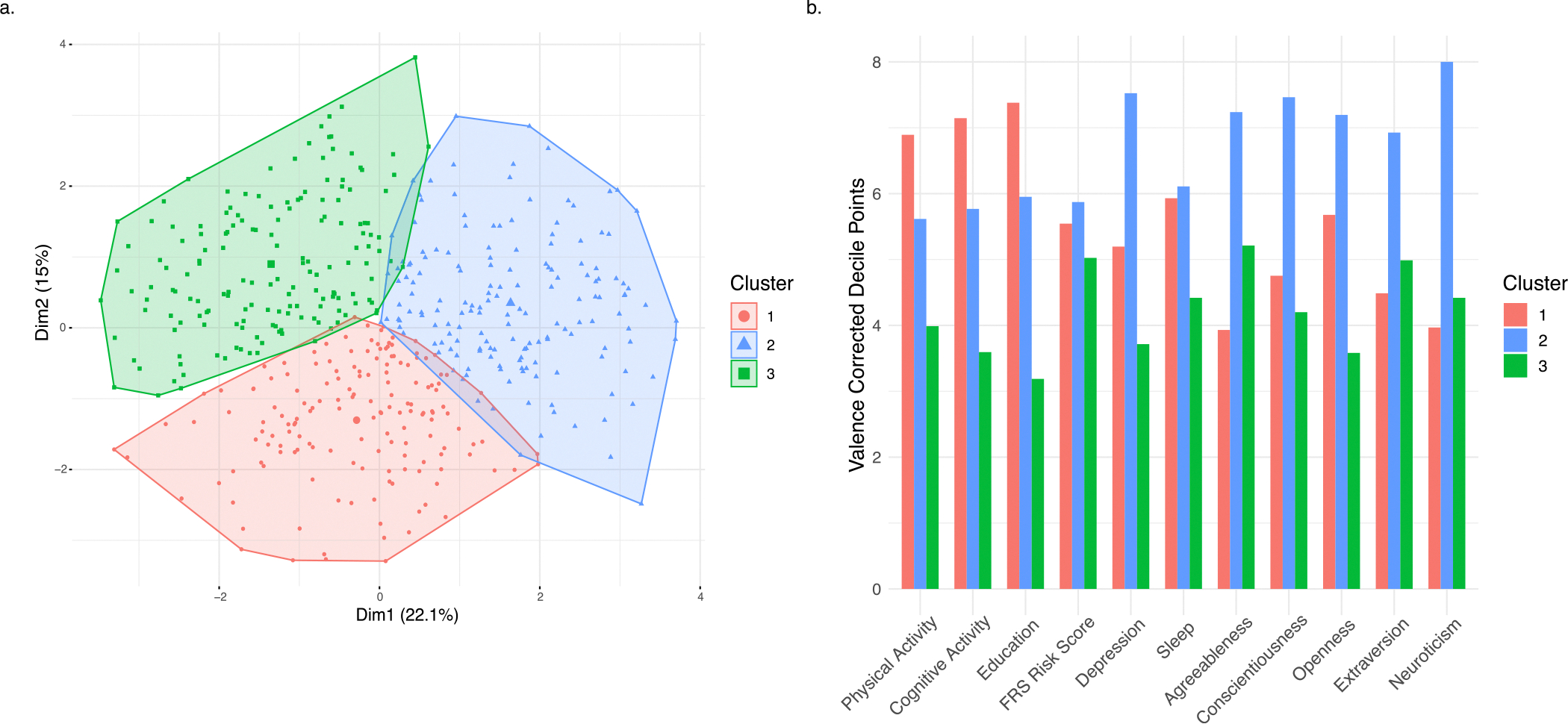
K-means clustering defines 3 risk factor profiles. PCA plot (a) and bar plot (b) of unsupervised k-means clustering of modifiable risk factors. a) We assigned input data points to one of three clusters in k-means analysis and generated PCA plots to visualize cluster boundaries after dimensional reduction. b) Variables were converted to decile points and multiplied by −1 where appropriate so that higher scores indicate better outcomes in all the variables. Clusters 1 and 2 appear to have more favorable risk factor profiles, while Cluster 3 appears to have a less optimal risk factor profile (negative multi-domain). Cluster 1 shows lower risk in the physical activity, cognitive activity, and education domains (positive-active), while Cluster 2 shows less risk in the sleep, depression, and personality domains (positive-affective).

K-means clustering was conducted on longitudinal lifestyle testing sessions from 203 BACS participants. Risk factor variables obtained from these sessions were converted into decile scores and valence corrected so that higher scores always indicated better outcomes prior to clustering. While principle component analysis (PCA) demonstrated a high degree of continuity between data points (Fig. 1a), we ultimately chose to use 3 clusters that revealed differential patterns of risk factor interrelationships in our dataset. Full descriptive risk factor data by cluster is reported in Supplementary Table 1. Significantly, our main findings from subsequent LMEMs remained consistent when using 2 or 4 clusters (Supplementary Fig. 1).

Each of the 3 clusters corresponded to a different profile of risk factor interrelationships, and participants were allocated to a profile based on the cluster assigned to the majority of their longitudinal sessions. Interestingly, clusters 1 and 2 both qualitatively appeared to represent healthier risk factor profiles than cluster 3, but along different domains. Namely, participants in cluster 1 exhibited higher physical activity, cognitive activity, and education scores than those in clusters 2 and 3. Participants in cluster 2, on the other hand, exhibited lower depression and better sleep quality, and more typically “positive” personality scores than those in clusters 1 and 3 (Fig. 1b). Therefore, we tested how these groupings could probe dissociations in cognition-pathology relationships between positive and negative risk profiles, as well as between positive-active (cluster 1) and positive-affective (cluster 2) risk profiles.

### 3.2. Cohort characteristics by cluster

Table 1 shows demographic information for each cluster cohort at cognitive baseline. Although age was significantly different between clusters (p = 0.006), it was controlled for in all subsequent LMEMs, along with sex. Furthermore, the proportion of females (p = 0.78) as well as cognitive follow-up years (p = 0.57) and number of sessions (p = 0.17), did not differ significantly between clusters, and notably, the proportion of APOE ε4 carriers also did not vary significantly between clusters (p = 0.65), suggesting that genetic risk for AD was not significantly different between participant groups. ANOVA tests at cognitive baseline further revealed no significant cross-sectional differences in NM (F(2, 198)= 1.73, p = 0.18) or EM (F(2, 195)= 2.08, p = 0.13) between clusters. Interestingly, we also found no differences between clusters in the proportion of PiB-positive participants (p = 0.47), indicating that Aβ pathology did not vary significantly between clusters. Moreover, we observed no significant effect of cluster on global PiB-DVR (p = 0.29), EC tau (p = 0.25), or MetaROI tau (p = 0.64), and this remained the case after controlling for covariates. Based on these findings, we conclude that risk factor profiles do not affect the degree of AD pathology.

### 3.3. Longitudinal change in episodic memory and non-memory cognition do not vary by cluster assignment

**Fig. 2. |:**
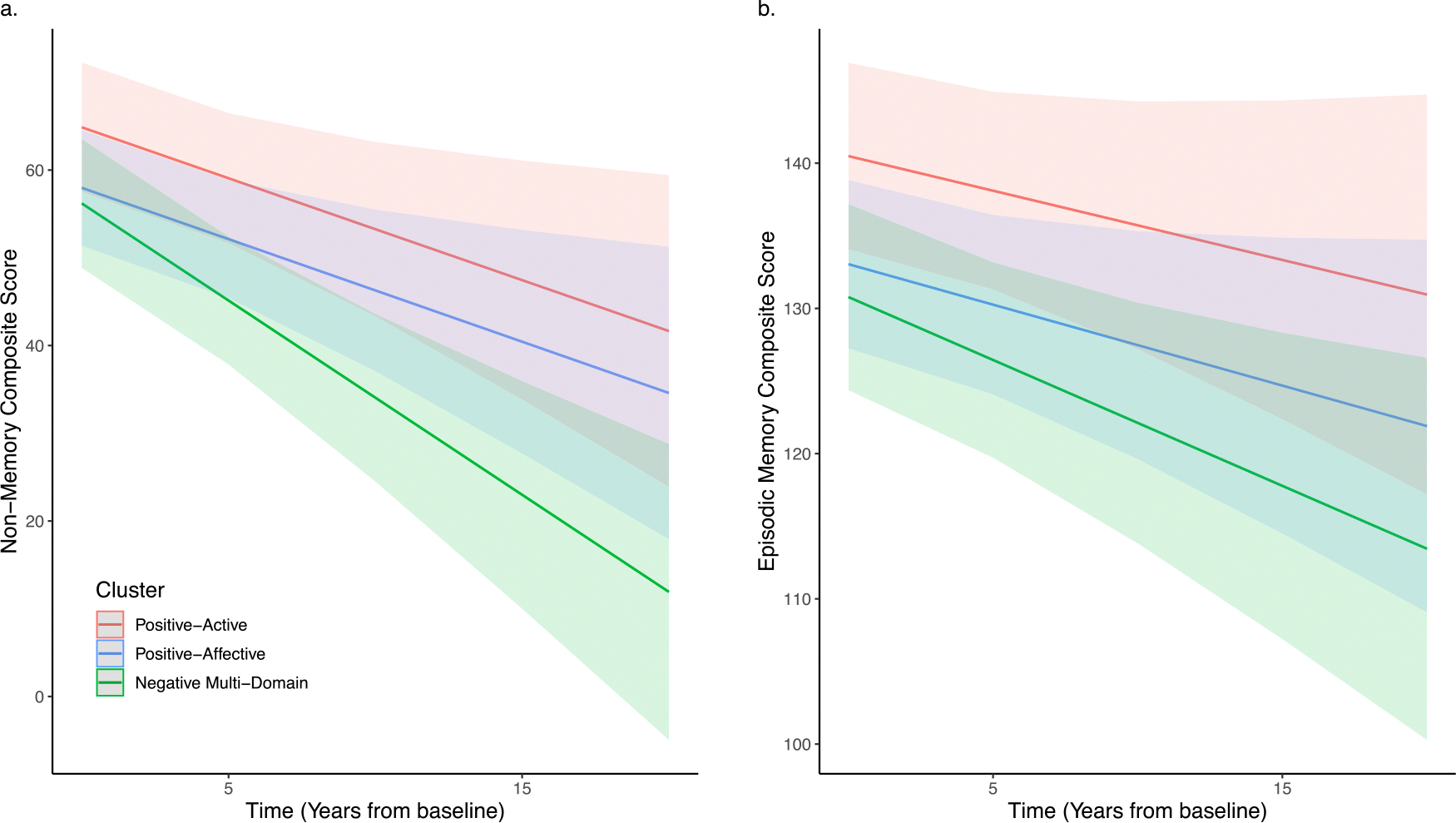
Longitudinal change in episodic memory and non-memory cognition do not vary by cluster assignment. Fitted longitudinal change in cognitive domain scores for non-memory cognition (a) and episodic memory (b) are plotted for each cluster. Shaded regions represent 95 % confidence intervals. No significant main or interactive effects of cluster on cognitive domain score are observed in either model. A significant effect of time indicates decline in both domains for all clusters. Follow-up comparisons revealed only a significant main effect difference between the positive-active vs negative multi-domain cluster in episodic memory.

In LMEMs predicting either NM or EM decline with age and sex as covariates (*cognition (NM or EM) ~ time* + cluster*time + *age* + *sex* + *(1* + *time | participant)*), we observed no significant main effects of cluster (NM: p = 0.22; EM: p = 0.09) or two-way interactions between cluster and time (NM: p = 0.17; EM: p = 0.66). Complete ANOVA parameters on the LMEMs are reported in Supplementary Table 2. However, when the negative multi-domain cluster was used as reference, comparisons between the positive-active and negative multi-domain cluster revealed a significant difference in the main effect on EM (p = 0.04; Table 2). To illustrate these relationships, we plotted both EM and NM domain scores within the context of each cluster assignment (Fig. 2).

### 3.4. Favorable risk factors attenuate the effect of Aβ, but not tau, on longitudinal cognitive decline

We hypothesized that healthier risk profiles would be associated with more favorable longitudinal cognitive outcomes and could potentially moderate the impact of AD pathology on cognitive decline.

#### 3.4.1. PiB-status

**Fig. 3. |:**
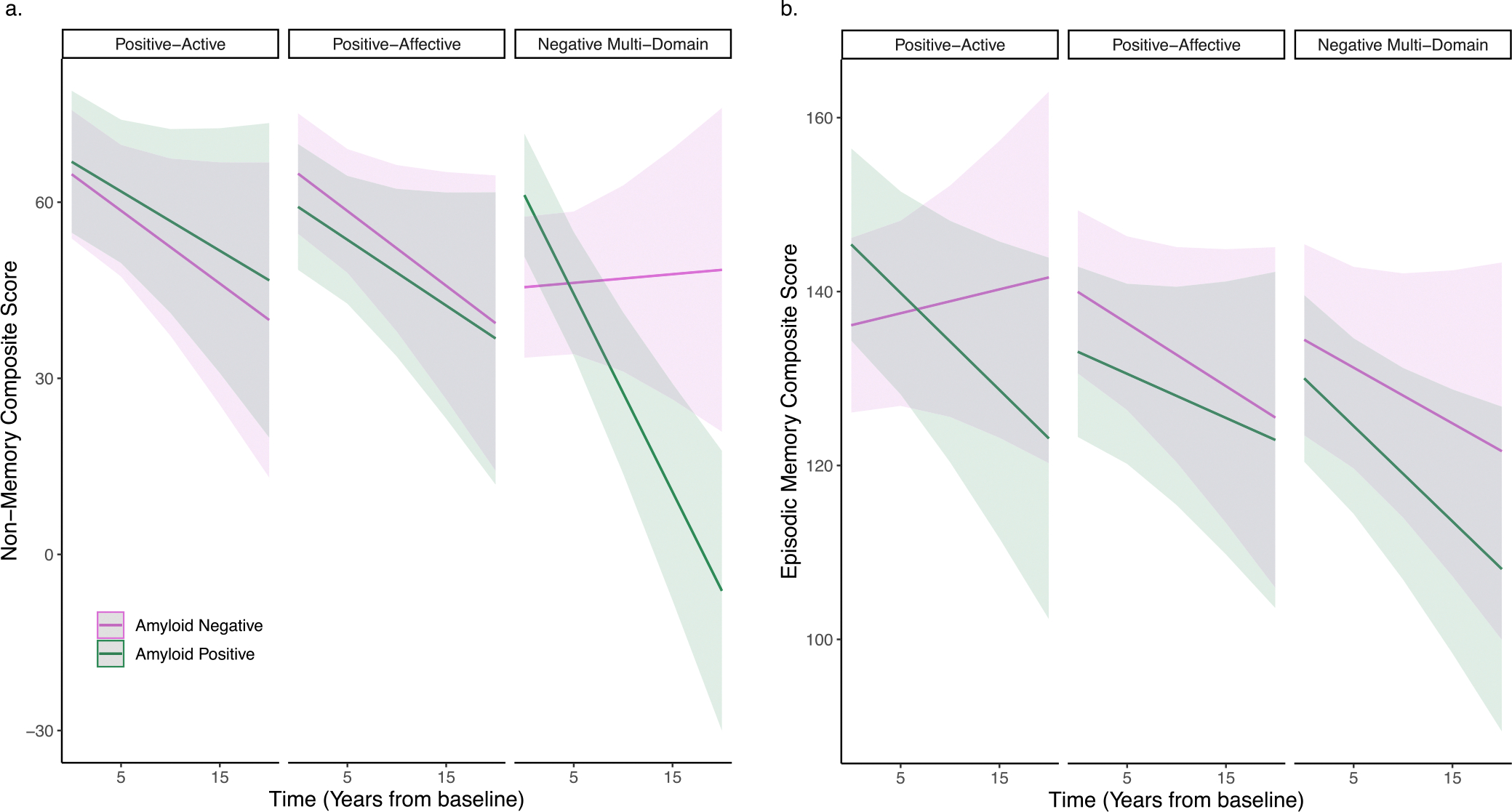
Cluster moderates the relationship between PiB-status and longitudinal decline in non-memory cognition. Fitted longitudinal change in cognitive domain scores for non-memory cognition (a) and episodic memory (b) are plotted for each cluster. Shaded regions represent 95 % confidence intervals. a) The plot reveals an attenuated effect of Aβ on non-memory cognition decline in positive-active (β=3.7, p = 0.008) and positive-affective (β=3.7, p = 0.007) clusters compared to the negative multi-domain cluster. b) The plot shows no significant effect of Aβ on episodic memory decline in positive-active (β=−0.9, p = 0.34) and positive-affective (β=0.7, p = 0.47) clusters compared to the negative multi-domain cluster.

In a LMEM predicting NM decline with PiB-status as the measure of AD pathology (*NM ~ time* + cluster*time + PiB-status*time + cluster*PiB-status*time + *age* + *sex* + *(1* + *time | participant)*), we observed significant main effects of age (p < 0.001) and cluster (p = 0.04), along with a noteworthy three-way interaction between PiB-status, cluster, and time (p = 0.008) (Supplementary Table 2). Between-cluster comparisons revealed higher NM scores in positive-active (β=19.2, p = 0.02) and positive-affective (β=19.3, p = 0.02) clusters compared to the negative multi-domain cluster, as well as an attenuated effect of PiB-status on NM decline in positive-active (β=3.7, p = 0.008) and positive-affective (β=3.7, p = 0.007) clusters compared to the negative multi-domain cluster (Table 3). Notably, these results were similar upon adding APOE ε4 carrier status or maximum EC tau as covariates to the model in sensitivity analyses, suggesting that the observed effect of cluster on the relationship between PiB-status and NM decline was independent of genetic risk or tau pathology (Supplementary). Our results also remained consistent when we replaced PiB-status with global PiB-DVR as the measure of AD pathology, supporting the notion that cluster interacts with Aβ pathology to influence cognition (Supplementary). In an additional sensitivity analysis designed to test if cluster assignment moderates the relationship between PiB-status and EC tau accumulation, we also found no significant interactive effects between PiB-status, cluster, and time, suggesting that the observed attenuation of Aβ-related NM decline in positive versus negative clusters was unrelated to differential Aβ-EC tau relationships (Supplementary). Conversely, our LMEM predicting EM with PiB-status (*EM ~ time* + cluster*time + PiB-status*time + cluster*PiB-status*time + *age* + *sex* + *(1* + *time | participant)*) revealed no significant main or interactive effects of PiB-status, cluster, and time (Supplementary Table 1). To illustrate the three-way interactions, we plotted the relationship between both NM and EM with PiB-status over time within the context of each cluster assignment (Fig. 3).

#### 3.4.2. Entorhinal Cortex Tau

**Fig. 4. |:**
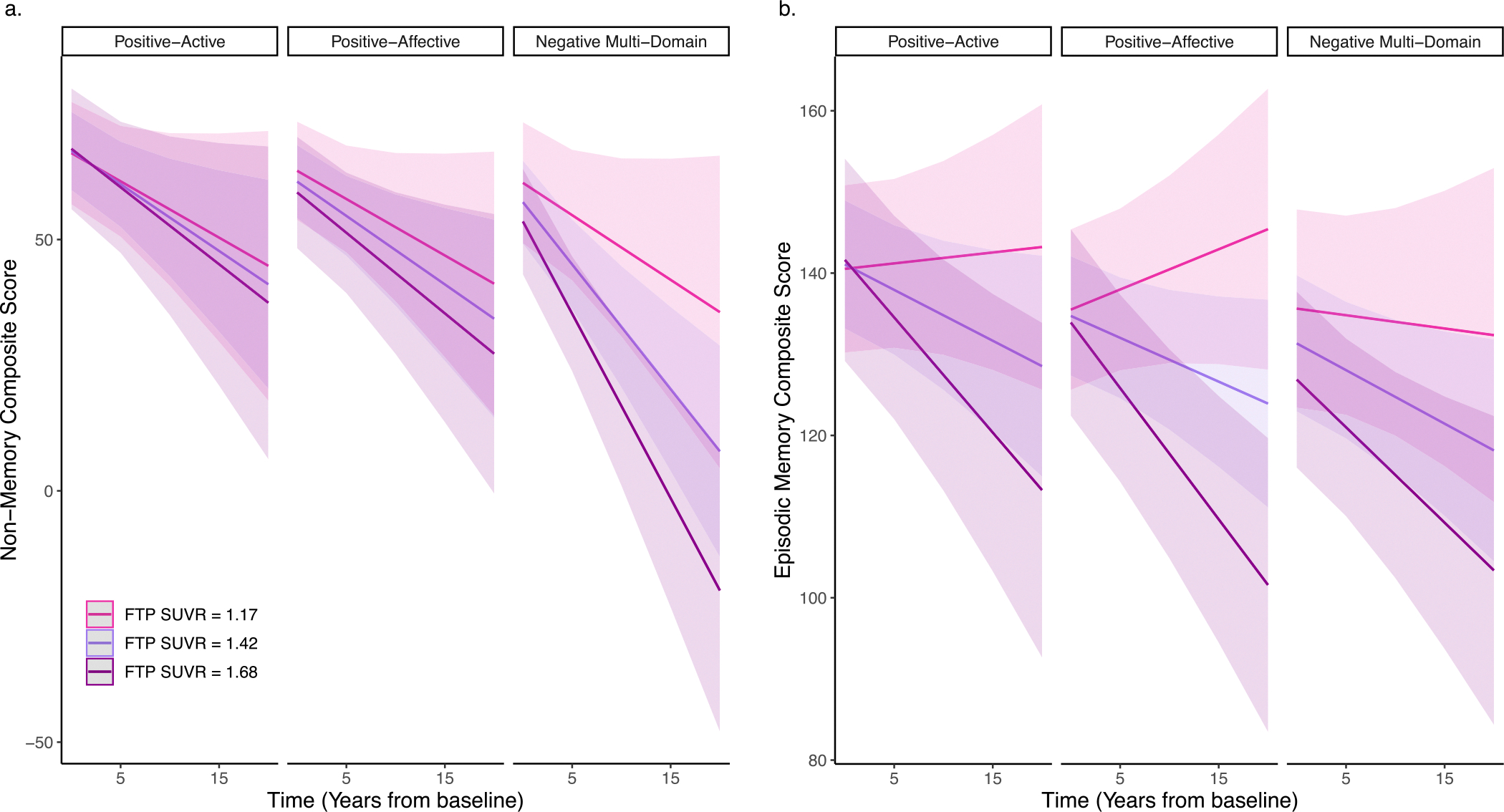
Cluster does not modify the relationship between entorhinal tau and longitudinal decline in episodic memory Fitted longitudinal change in cognitive domain scores for non-memory cognition (a) and episodic memory (b) are plotted for each cluster. Shaded regions represent 95 % confidence intervals. The plot shows no significant effect of cluster on a) entorhinal tau x non-memory cognition decline in positive-active (β=3.9, p = 0.19) or positive-affective (β=3.8, p = 0.19) clusters compared to the negative multi-domain cluster or b) entorhinal tau x episodic memory decline in positive-active (β=−1.1, p = 0.56) or positive-affective (β=−2.2, p = 0.26) clusters compared to the negative multi-domain cluster. There is a significant tau x time interaction (p < 0.001) on episodic memory.

In a LMEM predicting EM decline with EC tau as the measure of AD pathology (*EM ~ time* + cluster*time + EC tau*time + cluster*EC tau*time + *age* + *sex* + *(1* + *time | participant)*), we observed a significant main effect of age (p = 0.0002) as well as a significant EC tau x time interaction (p < 0.001) (Supplementary Table 2). However, this relationship was not modified by cluster assignment. Likewise, a LMEM predicting NM decline with EC tau as a predictor (*NM cognition ~ time* + cluster*time + EC tau*time + cluster*EC tau*time + *age* + *sex* + *(1* + *time | participant)*) demonstrated no significant main or interactive effects of EC tau, cluster, and time (Supplementary Table 1). To illustrate the three-way interactions, we plotted the relationship between both NM and EM with EC tau over time within the context of each cluster assignment (Fig. 4). These results were not modified by addition of PiB-status or APOE ε4 carrier status as covariates to either model, and were similar when substituting MetaROI tau for EC.

## 4. Discussion

In order to examine how intercorrelated risk factors and AD pathology influence cognitive decline, we developed risk factor profiles and examined how they moderated pathology-cognition relationships. While these risk factor profiles were not related to the presence or degree of Aβ or tau pathology, or cognitive decline across domains, Aβ positive individuals in the positive-active and positive-affective clusters had slower non-memory cognition decline than those in the negative multi-domain cluster. Our approach therefore revealed patterns of risk factor interrelationships as crucial cognitive reserve elements within Stern’s framework of resilience (Stern et al., 2023). As such, we propose that the patterns of risk factor interrelationships affect some property of the brain that enables cognitive performance to surpass expectations even in the presence of life-course accumulations of brain pathology (Stern et al., 2023)^.^

Our particular findings in non-memory cognition decline align with recent results from the Healthy Brain Project (HBP), which demonstrated that individuals with high multi-domain lifestyle risk showed worse cognitive performance in executive function and working/learning memory, but not in episodic memory (Bransby et al., 2024, 2023). Growing evidence from our cohort as well as others appears to indicate that beta-amyloid deposition affects a number of non-memory functions, including timed tasks and executive tasks (Chen et al., 2025; Farrell et al., 2022). While the mechanism for this is unclear, it may involve widespread cortical and particularly frontal effects; reflecting the distribution of amyloid as opposed to the medial temporal distribution of tau (Chen et al., 2025; Farrell et al., 2022), and potentially explaining the observed specificity of risk factor profiles in moderating the influence of Aβ, but not tau, deposition on cognition. Notably, however, our results also confirm the widely reported specific effects of medial temporal lobe tau on episodic memory decline (Chen et al., 2024; Hanseeuw et al., 2019; Sperling et al., 2019).

The specificity of our findings may also be attributed to a combination of factors. Results from the Harvard Aging Brain Study (HABS) cohort have indicated specific effects of Aβ pathology on non-memory cognition, with steeper PiB slopes being associated with declining processing speeds (Farrell et al., 2022). This finding along with our own observations, however, contrast results from the HBP that showed higher cerebrospinal fluid (CSF) levels of phosphorylated and total tau, but not Aβ, in the multi-domain risk group exhibiting executive function deficits (Bransby et al., 2024). Moreover, while the mechanisms linking risk factor profiles to the Aβ pathology pathway are still unknown, our results appear to highlight the role of risk factor profiles as resilience factors that mitigate the effects of Aβ deposition rather than reduce it, given that the proportion of PiB-positive participants and global PiB-DVR was not significantly different between clusters. Although prior work in the BACS cohort has demonstrated that lifetime cognitive activity (Wirth et al., 2014) and physical activity (Pedrero-Chamizo et al., 2024) both reduce Aβ accumulation in APOE ε4 carriers, suggesting that individual risk factors may still play some role in the resistance pathway, our findings point to broader risk factor interrelationships as being more implicated with resilience. Additionally, given recent evidence from the Alzheimer’s Disease Neuroimaging Initiative (ADNI) showing that individual modifiable risk factors differentially influence cognitive resilience depending on amyloid or tau pathology, we further highlight the importance of exploring the effects of risk factor interrelationships on the AD pathway (Huszár et al., 2024).

A notable limitation of this study is that the BACS cohort is primarily composed of affluent, well-educated, White individuals, and therefore is relatively homogeneous with respect to multiple potential exposure variables such as socioeconomic position and race. This homogeneity, in combination with the potential for recall bias and subjective interpretation issues stemming from self-reported risk factor data, in turn limits our ability to draw definitive conclusions about specific risk factor profiles both outside and within the BACS cohort. We nonetheless hope our study inspires similar holistic risk factor studies in diverse cohorts, to both improve the generalizability of our findings as well as validate them in an independent sample. Despite the BACS cohort homogeneity, our finding that groups with high multi-domain risk were consistently more sensitive to the effects of Aβ on non-memory cognition in multi-cluster analyses (Supplementary Fig. 1) demonstrates that we can still draw important conclusions regarding the role of risk factor interrelationships as AD resilience factors.

Our analyses additionally showed that both favorable risk factor profiles (positive-active and positive-affective) provided similar levels of resilience relative to the negative multi-domain group, suggesting that there may be multiple pathways that protect against the effects of Aβ deposition on non-memory cognition. Further research into the mechanisms linking these groups of modifiable risk factors to biological aging pathways will thus be important for future studies, especially given that several of the relationships between the risk factors and biological outcomes studied here may be bidirectional, and we were limited in our ability to establish temporality. Significantly, the 2-year Finnish Geriatric Intervention Study to Prevent Cognitive Impairment and Disability (FINGER) randomized control trial demonstrated that multi-domain lifestyle interventions in older adults with dementia risk could improve cognitive functioning and reduce risk of decline, suggesting the validity of intervening at a multi-domain level even at later time points (Ngandu et al., 2015).

## 5. Conclusions

In summary, this study delved into the dynamics of multiple modifiable risk factors in shaping cognitive aging. Our exploration, while building upon existing literature focusing on individual risk factors, underscored the importance of examining the collective influence of these factors across longitudinal aging and highlighted the pivotal role of risk factor profiles as cognitive reserve elements in Alzheimer’s Disease research. Moving forward, understanding the mechanisms linking modifiable risk factors to biological aging pathways and replicating this study in diverse study cohorts will be crucial for translating this research into both effective clinical prevention strategies and downstream interventions. Overall, we are optimistic that our findings can contribute to strategic improvements in AD interventions at both the individual and population level.

## Supplementary Material

1

## Figures and Tables

**Table 1:** Cluster-Specific Cohort Characteristics.

	Cluster 1 (positive-active) n = 62	Cluster 2 (positive-affective) n = 77	Cluster 3 (negative multi-domain) n = 64	P

Age at baseline, years	71.77 ± 6.41	72.49 ± 5.83	75.22 ± 6.76	< 0.01^a^
Sex, female, n (%)	34 (55)	41 (53)	38 (59)	0.78
APOE ε4 (%)^b^	12 (27)	10 (19)	13 (25)	0.65
PiB+, n (%)^c^	20 (43)	26 (47)	27 (56)	0.47
EC tau, FTP uptake^d^	1.38 ± 0.25	1.38 ± 0.24	1.46 ± 0.27	0.25
MetaROI tau, FTP uptake^d^	1.33 ± 0.23	1.36 ± 0.15	1.37 ± 0.17	0.64
Cognitive follow-up, years	6.89 ± 5.01	6.19 ± 4.83	6.95 ± 4.44	0.57
Cognitive sessions, n	5.34 ± 3.59	4.96 ± 3.49	6.06 ± 3.36	0.17

PiB: [^11^C]Pittsburgh Compound B, EC: entorhinal cortex, FTP: [18 F] flortaucipir, MetaROI: metatemporal region of interest. Values are expressed as mean ± sd unless otherwise specified. One-way ANOVA tests were used to calculate P values for age, years of education, EC tau, MetaROI tau, cognitive follow-up, and cognitive sessions. Fisher’s Exact tests were used to calculate P values for sex, PiB+, and APOE ε4 Carrier.

a Post-hoc Tukey’s honest significant difference (HSD) comparison of between-cluster differences were as follows. 1 vs 2: p = 0.78; 1 vs 3: p = 0.007; 2 vs 3: p = 0.03

b Participants were characterized according to presence (APOE ε2/APOE ε4, APOE ε3/APOE ε4, or APOE ε4/APOE ε4) or absence of an APOE ε4 allele. The number of participants with missing APOE ε4 data were as follows: Cluster 1: 18/62; Cluster 2: 15/77; Cluster 3: 13/64.

c The number of participants with no available PiB scans were as follows: Cluster 1: 21/62; Cluster 2: 30/77; Cluster 3: 28/64

d The number of participants with no available FTP scans were as follows: Cluster 1: 16/62; Cluster 2: 22/77; Cluster 3: 16/64. EC and MetaROI tau were characterized as the maximum FTP uptake recorded in longitudinal scans. Age- and sex-adjusted p-values are as follows: EC tau: p = 0.71, MetaROI tau: p = 0.80.

**Table 2:** Linear mixed-effects models parameter estimates: Predicting longitudinal cognitive decline with cluster assignment.

	Non-Memory Cognition			Episodic Memory		
			
Parameter	Estimate	s.e.	P	Estimate	s.e.	P

(Intercept)	183.6	24.3	< 0.001	240.6	22.0	< 0.001
Age	−1.8	0.3	< 0.001	−1.5	0.3	< 0.001
Sex (male ref)	7.4	4.0	0.06	4.2	3.6	0.24
Time	−2.2	0.4	< 0.001	−0.9	0.3	< 0.01
Cluster 1	8.7	5.4	0.11	9.7	4.7	0.04
Cluster1*Time	1.1	0.7	0.11	0.4	0.5	0.40
Cluster 2	1.8	5.1	0.73	2.3	4.4	0.61
Cluster2*Time	1.0	0.6	0.10	0.3	0.4	0.49

s.e., standard error. Longitudinal change cognition (non-memory and episodic memory) was assessed using linear mixed effects models. Cluster 1 and Cluster 2 refer to the positive-active and positive-affective clusters, respectively. Estimates are reported with cluster 3 (negative multi-domain) serving as the reference group.

**Table 3:** Linear mixed-effects models parameter estimates: Predicting longitudinal cognitive decline with cluster assignment and PiB-status.

Parameter	Non-Memory Cognition		P	Episodic Memory		
	Estimate	s.e.		Estimate	s.e.	P

(Intercept)	151.6	29.3	< 0.001	254.7	27.6	< 0.001
Age	−1.5	0.4	< 0.001	−1.7	0.3	< 0.001
Sex (male ref)	6.3	4.5	0.16	0.9	4.2	0.83
Time	0.1	0.7	0.84	−0.6	0.5	0.21
PiB-status	15.7	8.1	0.05	−4.4	7.4	0.55
PiB-status*Time	−3.5	1.0	< 0.001	−0.5	0.7	0.50
Cluster 1	19.2	8.4	0.02	1.7	7.7	0.82
Cluster1*Time	−1.4	1.0	0.17	0.9	0.7	0.21
Cluster1*PiB-status	−13.6	11.7	0.25	13.7	10.7	0.20
Cluster1*PiB-status*Time	3.7	1.4	< 0.01	−0.9	1.0	0.34
Cluster 2	19.3	8.2	0.02	5.5	7.5	0.46
Cluster2*Time	−1.4	1.0	0.15	−0.1	0.7	0.91
Cluster2*PiB-status	−21.4	11.1	0.06	−2.5	10.2	0.81
Cluster2*PiB-status*Time	3.7	1.3	< 0.01	0.7	0.9	0.47

s.e.: standard error, PiB: [^11^C]Pittsburgh Compound B. Longitudinal change cognition (non-memory and episodic memory) adjusted for PiB-status was assessed using linear mixed effects models. Cluster 1 and Cluster 2 refer to the positive-active and positive-affective clusters, respectively. Estimates are reported with cluster 3 (negative multi-domain) serving as the reference group.

## Appendix A. Supporting information

Supplementary data associated with this article can be found in the online version at doi:10.1016/j.neurobiolaging.2025.05.001.

## Glossary

AD: Alzheimer’s Disease
ADNI: Alzheimer’s Disease Neuroimaging Initiative
Aβ: β-amyloid
ANOVA: Analysis of Variance
BACS: Berkeley Aging Cohort Study
BMI: Body Mass Index
CFA: Confirmatory Factor Analysis
CSF: Cerebrospinal Fluid
CT: Computerized tomography
CVLT: California Verbal Learning Test
DVR: Distribution Volume Ratio
EC: Entorhinal Cortex
EM: Episodic Memory
FINGER: Finnish Geriatric Intervention Study to Prevent Cognitive Impairment and Disability
FRS: Framingham Risk Score
FTP: [18 F] flortaucipir
GDS: Geriatric Depression Scale
HABS: Harvard Aging Brain Study
HBP: Healthy Brain Project
HSD: Honest Significant Difference
LBNL: Lawrence Berkeley National Laboratory
LDFR: Long Delay Free Recall
LEQ: Lifetime of Experiences Questionnaire
LIBRA: Lifestyle for Brain Health
LMEM: Linear Mixed-Effects Model
MMSE: Mini-Mental State Examination
NM: Non-Memory Cognition
PACC: Preclinical Alzheimer Cognitive Composite
PCA: Principal Component Analysis
PET: Positron Emission Tomography
PiB: [11 C]Pittsburgh Compound B
PSQI: Pittsburgh Sleep Quality Index
PVC: Partial Volume Correction
ROI: Region of Interest
SDFR: Short Delay Free Recall
SUVR: Standardized uptake value ratio
TMT: Trail Making Test
