# Supplementary material for: Modifiable risk factor profiles moderate the effect of β-amyloid pathology on cognition in aging
Source: Neurobiol Aging. Author manuscript; Available in PMC 2026 Jun 11. (PMC13257327; doi:10.1016/j.neurobiolaging.2025.05.001)
Supplement: 1 [file NIHMS2176201-supplement-1.docx]

**Supplementary Materials**

Supplementary Table 1: Cluster-Specific Cohort Characteristics

|  | **Cluster 1 (positive-active)**  **n=62** | **Cluster 2 (positive-affective)**  **n=77** | **Cluster 3**  **(negative multi-domain)**  **n=64** |
| --- | --- | --- | --- |
| Physical Activity (kCal/week) | 3001.69 ± 2017.60 | 2741.35 ± 2578.06 | 1500.04 ± 1097.29 |
| Late Life LEQ Score | 34.06 ± 5.31 | 31.61 ± 6.57 | 27.33 ± 6.06 |
| Education, years | 18.16 ± 1.52 | 17.44 ± 1.75 | 15.54 ± 1.85 |
| FRS | 17.84 ± 3.93 | 17.6 ± 3.73 | 19.06 ± 3.93 |
| GDS Score | 4.37 ± 3.19 | 2.04 ± 2.39 | 6.23 ± 4.15 |
| Global PSQI Score | 4.60 ± 2.83 | 3.97 ± 2.62 | 6.06 ± 3.29 |
| Conscientiousness, B5 Score | 33.02 ± 4.72 | 37.99 ± 4.19 | 32.41 ± 5.95 |
| Agreeableness, B5 Score | 33.03 ± 3.53 | 37.62 ± 3.93 | 35.3 ± 4.20 |
| Extraversion, B5 Score | 25.56 ± 5.05 | 29.82 ± 5.55 | 26.25 ± 6.12 |
| Neuroticism, B5 Score | 20.60 ± 4.38 | 14.64 ± 3.60 | 20.08 ± 4.64 |
| Openness, B5 Score | 37.61 ± 5.83 | 40.71 ± 5.87 | 34.02 ± 5.06 |

kCal: kilocalorie. LEQ: Lifetime Experiences Questionnaire. FRS: Framingham Risk Score. GDS: Geriatric Depression Scale. PSQI: Pittsburgh Sleep Quality Index. B5: Big 5 Inventory

**Linear mixed-effects models demonstrate that risk factor profiles interact specifically with Aβ status and non-memory cognition decline**

Supplementary Table 2: ANOVA parameters for linear mixed-effects models

|  | **Non-Memory Cognition** | | |  |  |  | **Episodic Memory** | |  |  |
| --- | --- | --- | --- | --- | --- | --- | --- | --- | --- | --- |
| **Parameter** | df | Sum sq | Mean sq | F | P |  | Sum sq | Mean sq | F | P |
| *Model 1/2: Cluster* |  |  |  |  |  |  |  |  |  |  |
| Age | 1 | 4661.4 | 4661.4 | 32.5 | <0.001 |  | 3616.4 | 3616.4 | 28.7 | <0.001 |
| Sex (male ref) | 1 | 495.0 | 495.0 | 3.6 | 0.06 |  | 171.2 | 171.2 | 1.4 | 0.24 |
| Cluster | 2 | 428.7 | 214.3 | 1.5 | 0.22 |  | 604.2 | 302.1 | 2.4 | 0.09 |
| Time | 1 | 4731.4 | 4731.4 | 33.0 | <0.001 |  | 1471.1 | 1471.1 | 11.7 | <0.001 |
| Cluster*Time | 2 | 511.8 | 255.9 | 1.8 | 0.17 |  | 103.5 | 51.8 | 0.41 | 0.66 |
| *Model 3/4: PiB status* |  |  |  |  |  |  |  |  |  |  |
| Age | 1 | 2227.7 | 2227.7 | 16.3 | <0.001 |  | 2712.7 | 2712.7 | 22.1 | <0.001 |
| Sex (male ref) | 1 | 275.6 | 275.6 | 2.0 | 0.16 |  | 5.6 | 5.6 | 0.05 | 0.83 |
| Cluster | 2 | 932.8 | 466.4 | 3.4 | 0.04 |  | 75.6 | 37.8 | 0.3 | 0.74 |
| Time | 1 | 523.8 | 523.78 | 3.8 | 0.05 |  | 197.0 | 197.0 | 1.6 | 0.21 |
| Cluster*Time | 2 | 356.7 | 178.4 | 1.3 | 0.27 |  | 297.5 | 148.8 | 1.2 | 0.30 |
| PiB status | 1 | 105.9 | 105.9 | 0.8 | 0.38 |  | 3.2 | 3.2 | 0.03 | 0.87 |
| PiB status*Time | 1 | 485.3 | 485.3 | 3.6 | 0.06 |  | 241.8 | 241.8 | 2.0 | 0.16 |
| Cluster*PiB status | 2 | 508.7 | 254.3 | 1.9 | 0.16 |  | 340.7 | 170.3 | 1.4 | 0.25 |
| Cluster*PiB status*Time | 2 | 1365.6 | 682.8 | 5.0 | <0.01 |  | 351.3 | 175.7 | 1.4 | 0.24 |
| *Model 5/6: EC tau* |  |  |  |  |  |  |  |  |  |  |
| Age | 1 | 1996.9 | 1996.9 | 0.7 | <0.001 |  | 1935.3 | 1935.3 | 15.3 | <0.001 |
| Sex (male ref) | 1 | 823.0 | 823.0 | 5.8 | 0.02 |  | 204.5 | 204.5 | 1.6 | 0.21 |
| Cluster | 2 | 27.8 | 13.9 | 14.0 | 0.91 |  | 46.6 | 23.3 | 0.2 | 0.83 |
| Time | 1 | 95.3 | 95.3 | 0.7 | 0.42 |  | 1685.9 | 1685.9 | 13.4 | <0.001 |
| Cluster*Time | 2 | 196.7 | 98.4 | 0.7 | 0.50 |  | 203.2 | 101.6 | 0.8 | 0.45 |
| EC tau | 1 | 91.5 | 91.5 | 0.6 | 0.42 |  | 52.1 | 52.1 | 0.4 | 0.52 |
| EC tau*Time | 1 | 494.0 | 494.0 | 3.5 | 0.07 |  | 2353.1 | 2353.1 | 18.7 | <0.001 |
| Cluster*EC tau | 2 | 84.1 | 42.0 | 0.3 | 0.74 |  | 99.4 | 49.7 | 0.4 | 0.68 |
| Cluster*EC tau*Time | 2 | 326.4 | 163.2 | 1.1 | 0.32 |  | 197.5 | 98.8 | 0.8 | 0.46 |

PiB: [11C] Pittsburgh Compound B, EC: entorhinal cortex. Longitudinal change cognition (non-memory and episodic memory) (Model 1/2), adjusted for PiB status (Model 3/4), or EC tau (Model 5/6) was assessed using type III ANOVA models. All models were adjusted for age and sex.

**The attenuating effect of favorable risk factor profiles on Aβ-related cognitive decline remains after sensitivity analyses and is unlikely to be related to differential Aβ-EC tau relationships**

Three-way interactions between cluster, PiB-status, and NM decline in our LMEM (*NM ~ time + cluster*time + PiB-status*time + cluster*PiB-status*time + age + sex + (1 + time | participant)*) remained significant after adding APOE ε4 carrier status (F(2, 98.89), p=0.01) or maximum entorhinal tau (F(2, 83.89), p=0.03) as covariates. Moreover, the greater resilience of both the positive-active and positive-affective clusters relative to the negative multi-domain cluster to the effect of PiB-status on NM decline remained after controlling for APOE ε4 carrier status (positive-active: β=3.8, p<0.009; positive-affective: β=3.6, p=0.02) or maximum entorhinal tau (positive-active: β=3.7, p=0.02; positive-affective: β=3.7, p=0.01). This suggests that the resilience to Aβ pathology observed in healthy risk factor profiles relative to unhealthy ones is unaffected by genetic risk or tau pathology.

Furthermore, when we substituted PiB-status in our LMEM with PiB-DVR (*NM ~ time + cluster*time + PiB-DVR*time + cluster*PiB-DVR*time + age + sex + (1 + time | participant)*), we observed the same significant three-way interaction between cluster, Aβ pathology, and NM decline (F(2, 104.66), p=0.01), as well as the same increased Aβ resilience in positive-active (β=3.1, p=0.006) and positive-affective (β=3.0, p=0.04) clusters relative to the negative multi-domain cluster. This strengthens our finding that risk factor profiles specifically moderate the relationship between Aβ pathology and NM decline.

Finally, in a LMEM predicting EC tau accumulation with cluster assignment and PiB-status as the main interactive effects of interest (*EC tau ~ time + cluster*time + PiB-status*time + cluster*PiB-status*time + age + sex + (1 + time | participant)*), we observed no moderating effect of cluster on the relationship between Aβ and tau pathology. This suggests that the observed moderating effect of cluster on amyloid-cognition relationships is likely unrelated to differential Aβ-EC tau relationships between clusters.

**High-risk groups that show lower resilience to the effects of Aβ status on longitudinal cognitive decline can be isolated in k-means analyses with 2 and 4 clusters**

Similar to our 3-cluster k-means analysis, PCA plots for 2- or 4-cluster analyses demonstrated low separation between groups due to the continuity between input data points (Supplementary Fig. 1a and 1d). Nevertheless, we found that we were consistently able to isolate a negative multi-domain cluster, i.e. cluster 2 in our 2-cluster analysis and cluster 4 in our 4-cluster analysis (Supplementary Fig. 1b and 1e), suggesting that there were salient differences between low-risk versus high-risk profiles that could be captured across analyses. Importantly, these profile differences also consistently translated to differential resilience capacity to the effect of Aβ pathology on NM decline (Supplementary Fig. 1c and 1f). Indeed, ANOVA parameters for LMEMs corrected for baseline age and sex showed significant three-way interactions between cluster, PiB-status, and NM decline when we used either 2 clusters (F(1, 103.07), p=0.03) or 4 clusters (F(3, 104.60), p=0.009). Furthermore, in both cases, healthier profiles showed significantly greater attenuation of the effects of PiB-status on NM decline relative to unhealthy profiles, with cluster 1 (β=2.4, p=0.03) showing a greater resilience effect than cluster 2 in our 2-cluster model, and clusters 1 (β=3.5, p=0.01), 2 (β=4.5, p=0.003), and 3 (β=4.1, p=0.009) showing greater resilience effects than cluster 4 in our 4-cluster model. Also similar to the 3-cluster results reported in the main text, we found that significant interactions between EC tau and longitudinal EM decline were not moderated by cluster in either our 2-cluster or 4-cluster analysis. Overall, these results highlight that there are meaningful differences in multi-domain risk levels within the BACS that in turn influence pathology-cognition relationships.


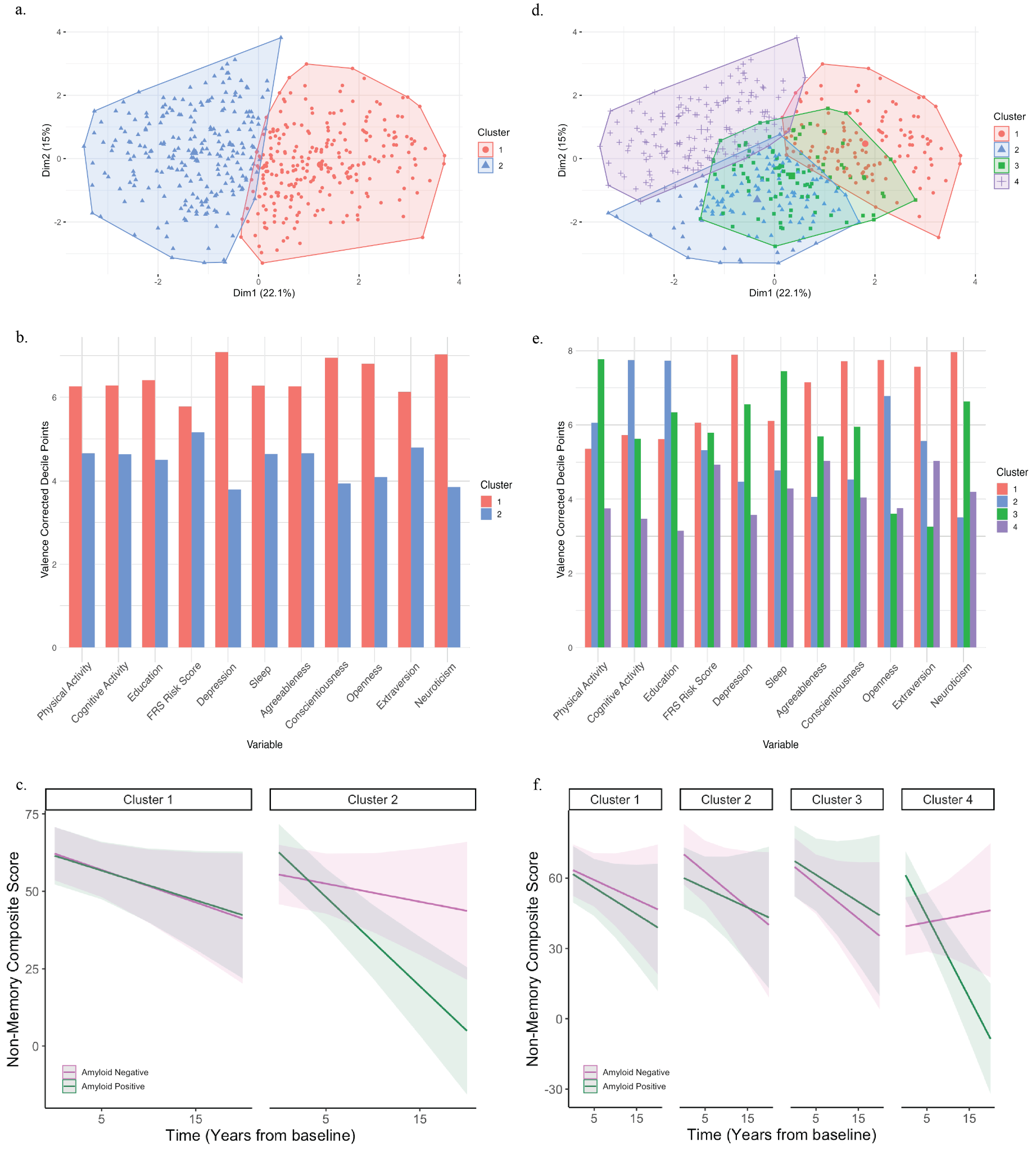


**Supplementary Fig. 1 | High-risk groups that show lower resilience to the effects of Aβ status on longitudinal cognitive decline can be isolated in k-means analyses with 2 and 4 clusters.** PCA plots (a, d) and bar plots (b, e) are shown for unsupervised k-means clustering of modifiable risk factors. We assigned input data points to one of two (a) or four (d) clusters in k-means analysis and generated PCA plots to visualize cluster boundaries after dimensional reduction. b, e) Variables were converted to decile points and multiplied by -1 where appropriate so that higher scores indicate better outcomes in all the variables. Cluster 1 appears to have a more favorable risk factor profile than cluster 2 (b), while clusters 1, 2, and 3 appear to have more favorable risk factor profiles than cluster 4 (e). Fitted longitudinal change in cognitive domain scores for non-memory cognition are plotted for each cluster in both our 2 (c) and 4 (f) cluster analyses. Shaded regions represent 95% confidence intervals. c) The plot reveals an attenuated effect of Aβ on non-memory cognition decline in cluster 1 (β=2.4, p=0.03) compared to cluster 2. f) The plot reveals an attenuated effect of Aβ on non-memory cognition decline in clusters 1 (β=3.5, p=0.01), 2 (β=4.5, p=0.003), and 3 (β=4.1, p=0.009) compared to cluster 4.
